# Microplastic Pollution in Pavement Sediments of Beijing: Abundance, Characteristics, and Ecological Risk

**DOI:** 10.3390/toxics14040323

**Published:** 2026-04-13

**Authors:** Donghai Yuan, Peinan Ye, Chenling Yan, Lili Xu, Jinggang Wang, Chen Wang, Ruiying Wu, Jun Cui, Yingying Kou

**Affiliations:** 1 Key Laboratory of Urban Stormwater System and Water Environment, Ministry of Education, Beijing University of Civil Engineering and Architecture, Beijing 100044, China; yuandonghai@bucea.edu.cn (D.Y.); ypnneil@outlook.com (P.Y.); sdtcwyh@163.com (R.W.); 2 Beijing Key Laboratory of Municipal Solid Waste Detection Analysis and Evaluation, Beijing Municipal Institute of City Management, Beijing 100028, China; yanchenling@aliyun.com; 3 China Railway Construction Network Information Technology Co., Ltd., Beijing 100043, China; 13426478876@163.com; 4 School of Information Technology, Nanchang Vocational University, Nanchang 330007, China; jingg-wang@163.com; 5 CAUPD (Beijing) Planning & Design Consultants Co., Ltd., Beijing 100044, China; sdjnkyy@163.com; 6 College of Chemical Engineering, Beijing University of Chemical Technology, Beijing 100029, China; cuijun900213@163.com

**Keywords:** microplastics, pavement sediments, risk assessment, Beijing

## Abstract

Pavement sediments are a significant source of microplastics (MPs) in urban environments and a major contributor to stormwater runoff pollution. In this study, we investigated the abundance and characteristics of microplastics in Beijing’s major road networks and functional zones by collecting road sediment samples, and assessed the ecological risks posed by microplastics in road surface sediments in Beijing. The results showed that the average abundance of microplastics in Beijing pavement sediments was 960.9 items/kg, with the highest abundance observed in commercial areas (1505.7 items/kg). The main characteristics of microplastics were black in color (22.4%), fibrous in shape (55.29%), small to medium in size (10–500 µm, 46.95%), with polyethylene (PE) being the most abundant polymer type (30.69%). The Polymer Risk Index (*PRI*) method showed clearer differentiation of spatial risk patterns in the Beijing study area, with 5 low-risk sites, 8 medium-risk sites and 15 high-risk sites among the sampling sites.

## 1. Introduction

Since the 1950s, global plastic production has increased exponentially, reaching 390 million tons in 2021 and projected to approach 33 billion tons annually by 2050. As the world’s largest producer, China contributed 32% of the global total in 2021 [1,2]. As emerging pollutants, microplastics are now widespread in oceans, rivers, soil, and the atmosphere and other environmental media [3,4], and have become a pressing global environmental issue due to their potential ecological and health risks. Microplastics, defined as plastic particles and fragments with a diameter of less than 5 mm [5], are a type of heterogeneous particle of plastics characterized by small size, easy access to living organisms, large specific surface area, and adsorption of pollutants, compared to bulky plastic wastes [6], and are more hazardous to the ecosystem [7]. As a megacity undergoing rapid urbanization, Beijing inevitably faces a variety of environmental challenges, among which microplastic pollution is emerging as a critical concern.

Although the initial attention on microplastics was focused on those in marine ecosystems, increasing attention has recently been directed toward their presence in urban environments. For example, the abundance of microplastics in road sediments in Chennai, India, was reported as 2279.4 ± 913.7 items/kg [8]; In Tokyo, Japan, the corresponding value was 230 ± 50 items/kg [9] and 35 ± 2 items/kg were found in wetland park sediments in Nanjing, China [10]. These findings indicate that pavement sediments are a major source of microplastics in stormwater runoff [11]. However, due to the complexity of microplastic isolation and detection, research on this topic remains limited [9]. The microplastics in pavement sediments primarily come from the degradation of automobile tires, road-marking paints and polymer-modified asphalt [12]. The characteristics and quantities of these pollutants depend on the road’s surroundings [13]. A study by Dehghani et al. showed that the average annual microplastic emissions onto roads, per resident, in Tehran, amounted to 1063–3223 per year [13]. Among various land-based pollution sources (such as tourism, rivers, transportation of trash and debris through air, and wastewater discharges), stormwater runoff is a major source of pollutants in open waters [14]. The presence of 15–38% synthetic rubber in stormwater treatment wetlands indicates that vehicle tires contribute to microplastic pollution of water and that stormwater runoff is an important pathway for microplastics in road sediments to enter the water environment [15].

Due to their environmental persistence, microplastics tend to accumulate in sediments, thereby elevating the ecological risks within these environments [16]. Therefore, assessing ecological risks and identifying pollution sources are essential for the effective management of microplastics in pavement sediments. At present, research on the risk assessment of microplastics remains in its early stages, and both domestic and international researchers have explored the environmental risks of microplastic pollution in different regions based on various assessment models [16,17,18,19]. This study investigates the current status of microplastic pollution in pavement sediments in Beijing, enriches the spatial distribution data of microplastics in megacities. This study also analyzes the morphological characteristics and polymer types of microplastics and further evaluates their potential environmental risks. The findings aim to provide a scientific basis for the prevention and control of microplastic pollution in megacities.

## 2. Materials and Methods

### 2.1. Study Area and Sample Collection

Beijing, a megacity with a resident population of 21.84 million and 16 municipal districts under its jurisdiction with high functional area integrity, is China’s political, economic, international communication and scientific and technological innovation center. The urban structure of central Beijing follows a north–south and east–west axis layout, with Tiananmen Square as the geographic center, a Chang’an Avenue and its extension as the horizontal axis in the east–west direction, and in the north–south direction, from Tiananmen Square, respectively, to the north to reach the Gulou Street until the Olympic Forest Park, forming the North Axis; to the south through the Yongdingmen Gate to reach the South Axis intersection to form the South Axis, which is “a horizontal and a vertical”. This “a horizontal and a vertical” framework, combined with the concentric system of five ring roads, shapes Beijing’s layered urban structure. These rings encompass diverse functional areas, including residential, commercial, transportation, and scenic districts, providing a representative framework for analyzing spatial heterogeneity in microplastic pollution.

**Figure 1:**
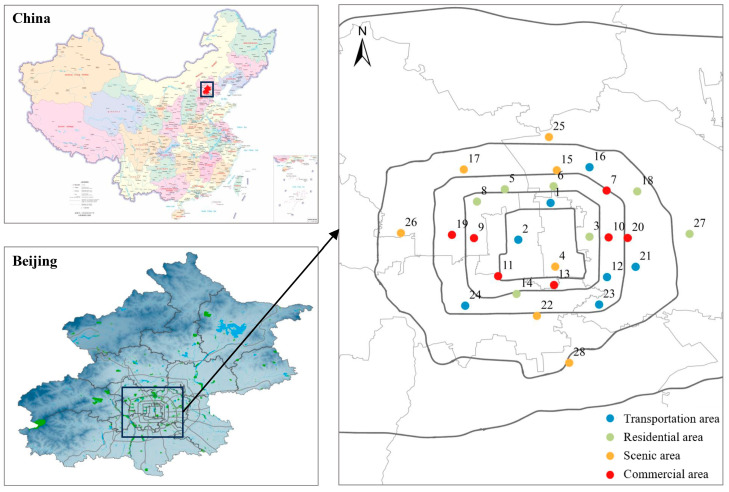
Location of the study area and distribution of the sampling points.

In this study, 28 sampling sites (Table A1 and Figure 1) were systematically distributed across the major ring roads and representative urban functional areas of Beijing, including residential, commercial, transportation, and scenic zones, to capture spatial differences in land use and human activity intensity. The sites covered the Second to Fifth Ring Roads, as well as the north–south central axis and Chang’an Avenue corridor, thereby providing broad spatial coverage of the central urban area. Sampling was conducted on sunny days with wind speeds below 1 m/s and no precipitation within the preceding three days. At each site, three random road sediment subsamples were collected and composited into one representative sample. After removing larger impurities, about 2 kg of sediment was transferred in its entirety to an aluminum foil bag using a clean iron dustpan, labeled, and transported to the laboratory within 24 h and stored in a refrigerator at 4 °C.

### 2.2. Sample Handling

The flotation method is widely used due to its accessibility and simple applicability to the studied materials [17]. A saturated NaCl solution (ρ = 1.20 g/cm^3^) (Guangfu Technology Development Co., Ltd., Tianjin, China) was selected as the flotation medium because it is widely used in microplastic extraction studies, accounting for approximately 45% of reported density separation methods, and offers advantages in terms of low cost, operational simplicity, and environmental friendliness [20]. Collected samples were first picked over to remove large impurities, then dried in a blast drying oven at 60 °C. The dried samples were sieved sequentially through stainless steel sieves of 5 mm (16 mesh), 2 mm (35 mesh), 1 mm (80 mesh), and 0.3 mm (250 mesh) and placed in centrifugal tubes. The solids on the 5 mm sieve were discarded, and the samples on the 2 mm and 1 mm sieves were observed under an ultra-fine microscope to isolate suspected microplastics, which were subsequently transferred to clean glass Petri dishes. Meanwhile, the material associated with the 0.3 mm fraction, including particles both retained on and passing through the 0.3 mm sieve, was collected in centrifuge tubes and subjected to density separation using the saturated NaCl solution. First, the samples were centrifuged at high speed for 30 min (centrifugation rate of 4000 rpm), and after the liquid level stabilized the supernatant was poured into a washed beaker, and then vacuum filtration, through a 50 mm glass-fiber filtration membrane of 0.22 µm pore size. After filtration, the membranes were immersed in 30% hydrogen peroxide solution (Tongguang Fine Chemicals Company, Beijing, China) and digested at room temperature for 48 h to remove organic matter, a second flotation was performed on the digested solution, following the same procedure as described above. After the secondary flotation, the solution was vacuum-filtered and subsequently dried in an oven at 60 °C until a constant weight was achieved, and the sample was stored for further observation.

### 2.3. Observation and Characterization of MPs

Microplastics retained on the filter membranes were observed and identified using a super depth-of-field digital microscope system combined with a laser confocal microscopic Raman spectrometer (HORIBA, LabRAM HR Evolution, Palaiseau, France), which was calibrated by a laser wavelength of 532 nm selected in the range of 300 cm^−1^ to 3200 cm^−1^. Suspected microplastics—including particles, films, fibers, and fragments—were visually identified based on their distinct colors, well-defined shapes, and reflective surfaces. For each suspected microplastic, physical characteristics including color, shape, and size were recorded and photographed, their Raman spectra measured, and the specific polymer type of the substance confirmed by comparing their Raman spectra with those of standard plastics. The abundance of microplastics was then estimated using particle-finding software, and microplastic abundance was measured in units of dry weight per kilogram of particles.

### 2.4. MPs Ecological Risk Assessment Methodology

#### 2.4.1. Pollution Load Index Method (*PLI*)

Pollutant loading indices are widely used to assess the abundance of pollutants released into the environment [18]. The method can simultaneously consider the environmental impacts of individual pollutants at a given location and the synergistic effects of multiple pollutants [21]. The criteria for classifying microplastic pollution load are as follows: low pollution load (*PLI* < 10), medium pollution load (10 ≤ *PLI* < 20), high pollution load (20 ≤ *PLI* < 30) and very high polymer risk (*PLI* ≥ 30).

The formula for calculating the microplastic pollution load index *PLI* is as follows:

(1) $$CF_{i}=\frac{C_{i}}{C_{0}}$$

(2) $$PLI_{i}=\sqrt{CF_{i}}$$

(3) $$PLI=\sqrt[{n}]{PLI_{1}\times PLI_{2}\times \cdots PLI_{n}}$$

where *CF_i_* is the pollution load factor of microplastics in the sediments at the sampling sites; *C_i_* is the abundance of microplastics in the sediments at the sampling sites; *C*_0_ is the background abundance of microplastics in the sediments on urban pavements. Due to a lack of available background data, the lowest MP concentration measured in this study was adopted as the background value. This approach is commonly adopted in cases where regional background values are unavailable, as it provides a conservative estimate for assessing relative pollution levels [22,23]; *PLI_i_* is the pollution load index of microplastics in the sediments at the sampling sites; and *PLI* is the pollution load index of microplastics in the sediments in the study area.

#### 2.4.2. Polymer Risk Index Method (*PRI*)

Polymer risks determined by the biosensitivity and biotoxicity of polymers and reflects the negative impacts of microplastics in sediments on surrounding organisms. The *PRI* is generally used to calculate the polymer risk of microplastics in sediments [24,25]. The calculation of *PRI* is proposed based on the Polymer Risk Scale [26], where *PRI* < 10 is low polymer risk, 10 ≤ *PRI* < 100 is moderate polymer risk, 100 ≤ *PRI* < 1000 is high polymer risk, and *PRI* ≥ 1000 is very high polymer risk. The formula for calculating *PRI* is as follows:

(4) $$PRI=\sum_{n=1}^{n}P_{i}\times S_{n}$$

where *P_i_* is the proportion of each polymer in the sediment at the sampling site; *S_n_* is the hazard score for each polymer (Table A2); and *PRI* is the pollution load index of microplastics in sediments at the sampling site.

#### 2.4.3. Potential Ecological Risk Index Method (*PERI*)

The potential ecological risk of microplastics considers the effects of abundance and polymer type on the ecological risk of microplastics [16]. Currently, *PERI* is commonly used to reflect the potential ecological risk of microplastics in sediments and is calculated using the following formula:

(5) $$C_{f}=\frac{C_{i}}{C_{0}}$$

(6) $$T_{i}=\sum_{n=1}^{n}\frac{P_{n}}{C_{i}}\times S_{n}$$

(7) $$PERI=T_{i}\times C_{f}$$

where *C_f_* is the enrichment factor of microplastics in the sediment at the sampling site; *C_i_* is the abundance of microplastics in the sediment at the sampling site; *C*_0_ is the background abundance of microplastics in the sediment; *T_i_* is the toxicity coefficient of the polymers; *P_n_* is the abundance of each polymer in the sediment at the sampling site; *S_n_* is the hazard score of each polymer; and *PERI* is the potential ecological risk index of microplastics in sediments at the sampling site.

Low potential ecological risk (I) was considered to exist when *PERI* < 150, medium potential ecological risk (II) when 150 ≤ *PERI* < 300, high potential ecological risk (III) when 300 ≤ *PERI* < 600, hazardous potential ecological risk (IV) when 600 ≤ *PERI* < 1200, and very hazardous potential ecological risk (V) when *PERI* ≥ 1200.

### 2.5. Statistical Analysis

Prior to hypothesis testing, the normality of the data was evaluated using the Shapiro-Wilk test, and homogeneity of variance was assessed using Levene’s test (The median microplastic abundances in scenic, transportation, residential, and commercial areas were 511.65, 545.76, 830.01, and 1326.50 items/kg, respectively. The corresponding interquartile ranges were 204.66, 1080.15, 1023.30, and 1114.06 items/kg). As the data did not meet the assumptions of normality and homogeneity, the non-parametric Kruskal–Wallis test was employed to assess differences in microplastic abundance among different functional regions. The latitude and longitude of each sampling point was determined using a GPS instrument. Spearman’s rank correlation analysis was used to examine the relationships between microplastic abundance, characteristics, and the three ecological risk indices (*PLI*, *PRI*, and *PERI*). The data were recorded using Microsoft Excel 2019. Data visualization was performed using Origin 2021 and ArcGIS 10.2 software. The results were analyzed using SPSS analysis software (28.0.1, IBM, Armonk, NY, USA).

### 2.6. Quality Control

To minimize potential contamination from airborne microplastics and other external sources, strict contamination control measures were implemented throughout the experimental procedures. Cotton laboratory coats were worn during all experimental operations. All instruments and glassware were thoroughly cleaned prior to use and subsequently wrapped in aluminum foil to prevent contamination from airborne deposition. Procedural blanks were included to monitor potential contamination during the experimental process. During the experiments, doors and windows were kept closed to minimize the introduction of contaminants carried by airflow.

## 3. Results

### 3.1. Abundance and Characterization of MPs in Different Loop Areas

#### 3.1.1. Abundance of MPs

**Figure 2:**
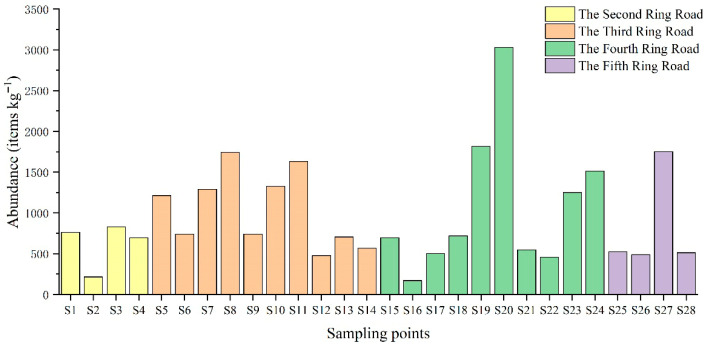
Abundances of microplastics in all the pavement sediment samples in the study area in Beijing.

Across all sampling sites, pavement sediments exhibited an average microplastic abundance of 960.9 items/kg (Figure 2). This is notably higher than in urban subsurface, where the average abundance typically ranges from tens to hundreds of items/kg [27,28]. In densely populated and highly urbanized areas, plastics are widely used in the daily activities of residents, which explains the high levels of microplastics in Beijing’s pavement sediments.

Microplastic abundances varied spatially across the ring roads, with averages of 625.4 ± 241.2, 1043.0 ± 429.9, 1069.9 ± 817.9, and 818.6 ± 538.4 items/kg for the Second to Fifth Ring Roads, respectively. The abundance of microplastics in all sampling sites ranged from 170.6 items/kg to 3032 items/kg, which is consistent with the range of results from previous studies of microplastics in Beijing roadside soils [29,30]. The spatial trend showed an increase from the Second to the Fourth Ring Road, followed by a decrease at the Fifth Ring Road, with the highest value observed at the Fourth Ring Road. The mean value of microplastic abundance on Chang’an Avenue and its extension was 1275.3 ± 854.2 items/kg, while the mean value of microplastic abundance on the north–south central axis was 635.3 ± 111.2 items/kg. This disparity may be associated with the commercial character of the Chang’an Avenue corridor. Intensive service activities and high traffic density in this area could contribute to elevated microplastic inputs, potentially through packaging-related waste and abrasion-derived particles [31]. High urban population density and intensive anthropogenic activities may represent the most important drivers of microplastic abundance in pavement sediments [32,33] and differences in microplastic abundance in different loops may be related to factors such as the urban economy, type of activity, and frequency of surface cleaning [34].

#### 3.1.2. Types of MPs

**Figure 3:**
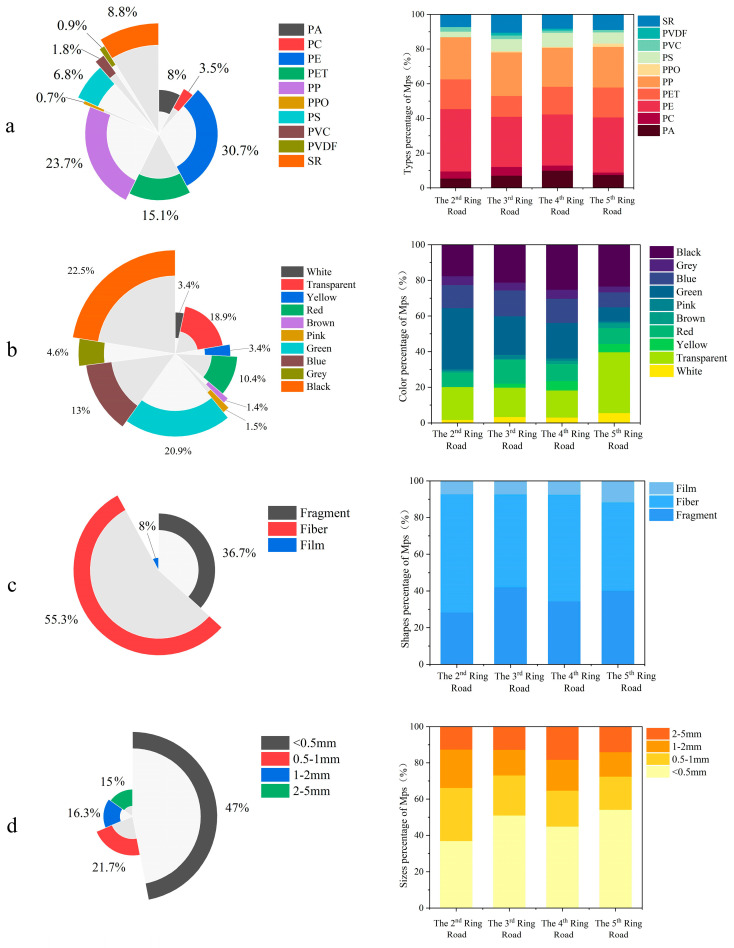
Distribution of (**a**) type, (**b**) color, (**c**) shape, and (**d**) size of microplastics in pavement sediments in Beijing and the four different ring road areas.

Based on Laser Raman confocal analysis, 1427 particles were confirmed as microplastics, accounting for 68% of all visually suspected particles analyzed, including 10 types, as shown in Figure 3a. Polyethylene (PE) accounted for the highest proportion (30.7%), followed by polypropylene (PP) with 23.7%, and polyethylene terephthalate (PET) with 15.1%. Silicone rubber (SR) and polystyrene (PS) were also detected but at lower proportions, this result shows similarity to the findings reported by Gu in prior research [35]. The distribution pattern of polymer types was generally consistent across samples from the Second to Fifth Ring Roads, PE was the most dominant polymer at all four ring locations, comprising 36.0%, 28.9%, 29.5%, and 31.8% of the total identified microplastics, respectively, PP ranked second, accounting for 24.3%, 25.1%, 22.5%, and 23.4%, respectively. Both PE and PP are widely used in food bags and food labels, and are also found in pots, pails, toys and plastic accessories used in everyday life [27]. PET and SR ranked third and fourth, respectively, in the percentage of detected microplastics, presumably due to the widespread use of PET in clothing, blankets, take-out food containers, and courier packaging [36,37], while SR is the more common type of polymer found in road deposits, as it originates from the wear and tear of automobile tires [38].

#### 3.1.3. Color, Shape and Size of MPs

The colors of microplastics in the pavement sediments in Beijing are shown in Figure 3b. The highest percentage was of black microplastics—22.5%, followed by green (20.9%), transparent (18.9%), blue (13.0%) and red (10.4%), with other colors accounting for smaller proportions. Across the ring roads, the dominant colors varied: in the Second Ring Road, green was most prevalent (34.2%), followed by transparent (18.5%) and black (17.6%); in the third ring road microplastics were similarly predominantly green (21.6%), followed by black (21.2%) and transparent (16.4%); in the fourth ring road zone the highest percentage was black (25.2%), followed by green (19.9%) and transparent (15.2%); in the fifth ring road zone, the share of transparent color microplastics (34.1%) was the highest, followed by black (23.4%). The high percentage of black microplastics in urban pavement sediments may be due to black debris from the wear and tear of automobile tires. The high percentage of colored microplastics may be due to the plastic content of building materials, toys, furniture and other plastic crushing, and colored microplastics are widely used in daily life because of their esthetic characteristics [37]. However, the color of microplastics will gradually change with external weathering, sunlight radiation and other effects [39], especially considering that they were from sediment samples in this study, indicating that some of the transparent microplastics may have originally been colored, but had faded over time, resulting in the large proportion of transparent microplastics found in this study.

**Figure A1:**
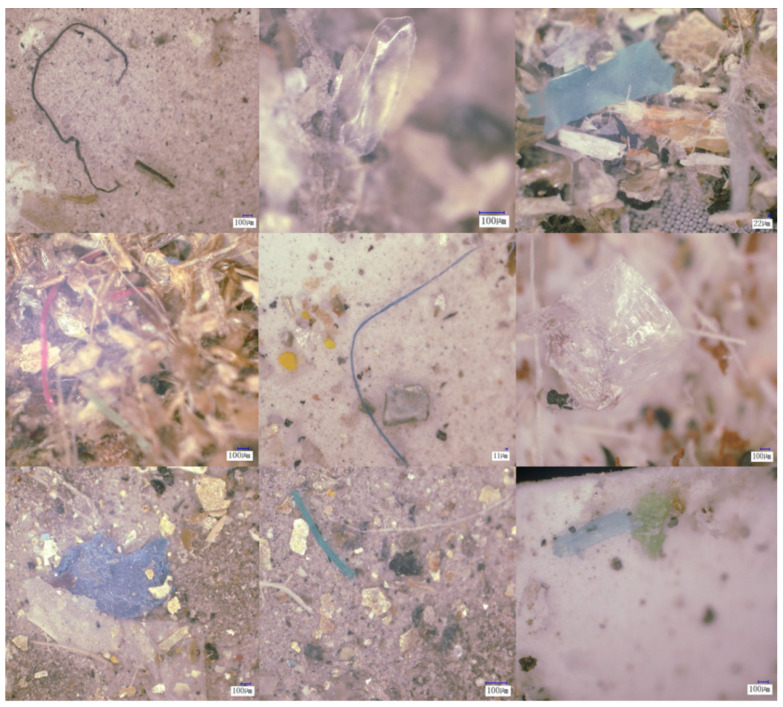
Microscopic photographs of typical microplastics in Beijing pavement sediments.

In our study, microplastics of different shapes and sizes were also detected (Figure 3c,d), and their morphological features under the microscope were photographed by ultrafine microscopy, as shown in Figure A1. Morphologically, fibers were the most prevalent type (55.3%), followed by fragments (36.7%) and films (8.0%), with fibrous microplastics dominating in all ring areas, possibly linked to disposable mask usage during the COVID-19 pandemic [40]. The shape of microplastics in the four different ring districts was predominantly fibrous, with 64.4%, 50.6%, 58.2%, and 48.1% of fibrous microplastics in Ring Roads 2 through 5, respectively. The shape of the microplastic with the lowest percentage in all four ring districts was in the form of a thin film, with 7.2%, 7.3%, 7.4%, and 11.7% in Ring Roads 2 through 5, respectively. Debris-like microplastics were in the middle of the list.

Microplastic sizes were classified into four categories: 10–500 µm, 0.5–1 mm, 1–2 mm, and 2–5 mm. The smallest size fraction (10–500 µm) accounted for the highest proportion (47.0%), followed by 0.5–1 mm (21.7%), 1–2 mm (16.3%), and 2–5 mm (15.1%). Based on 1427 identified particles, the average microplastic size in urban pavement sediments was 1013.2 µm. Across the four ring roads, microplastics smaller than 0.5 mm accounted for the highest percentages, with 36.9%, 51.0%, 44.9%, and 54.2% of microplastics smaller than 0.5 mm in the Second to Fifth Rings, respectively; microplastics in the range of 0.5–1 mm accounted for the next highest percentages, while those in the range of 1–2 mm and 2–5 mm were in the lowest percentages of microplastics in the several ring districts. This distribution is likely attributable to the degradation of larger plastics into smaller plastic fragments after entering the environment, via ultraviolet radiation, microorganism activities, etc. [41]. Relatively fewer small-sized microplastics (<0.5 mm) were accounted for in the second ring road. This pattern may be partly attributed to differences in street cleaning frequency across urban areas. In central urban zones, where population density, commercial activity, and municipal management intensity are generally higher, more frequent street cleaning can effectively remove larger particles [34]. In contrast, peripheral areas may receive less intensive maintenance, allowing greater accumulation of microplastics in sediments. These spatial disparities may also reflect underlying socioeconomic differences, which influence urban infrastructure investment and environmental management practices. Such differences could potentially lead to uneven exposure risks to microplastics among residents in different urban areas.

### 3.2. Abundance and Characterization of MPs in Different Functional Areas

#### 3.2.1. Abundance of MPs

**Figure 4:**
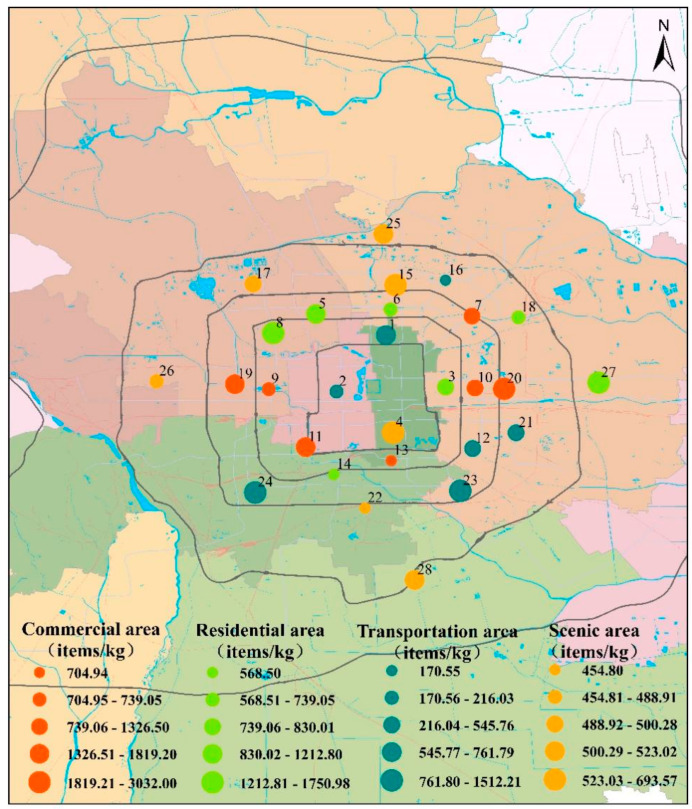
Distribution of microplastic abundance in pavement sediments in Beijing under different functional areas.

In this study, the mean abundances of microplastics in residential, transportation and scenic areas were 1080.7 items/kg, 704.9 items/kg, and 552.3 items/kg, respectively. In contrast, commercial areas showed a significantly higher average abundance of 1505.7 items/kg (Figure 4), likely due to the extensive use of plastics in stores and restaurants, which increases the potential for microplastics to enter the environment. This finding is consistent with previous study reporting higher microplastic concentrations in areas characterized by intensive street food activities, fast-food services, and the presence of shopping centers, where plastic consumption and waste generation are substantially elevated [29].

The results of the Kruskal–Wallis test indicated that microplastic levels were significantly higher in the commercial areas than in transportation areas and scenic areas (The *p* = 0.00172 and *p* = 0.03501, respectively, both <0.050). Intensive pedestrian activity, diverse pollution inputs, and the widespread use of plastic products may contribute to this pattern [9,42]. Residential areas, characterized by relatively lower foot traffic, moderate pollutant emissions, and intermediate cleaning frequency, exhibited correspondingly moderate levels of microplastic abundance. In contrast, transportation and scenic areas showed comparatively lower microplastic abundance, which may be associated with lower pedestrian activity intensity and shorter residence times, thereby reducing the opportunities for plastic debris to accumulate on road surfaces. Overall, both the abundance and composition of microplastics varied among different urban functional areas. Consistent with previous studies, our results showed that microplastic abundance in commercial areas was significantly higher than that in residential areas [43], suggesting that daily human activities play an important role in the generation and accumulation of microplastic pollution.

#### 3.2.2. Types of MPs

**Figure 5:**
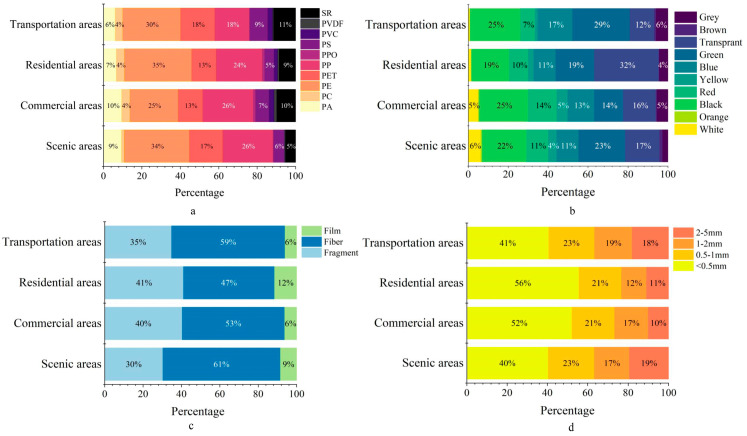
Distribution of (**a**) type, (**b**) color, (**c**) shape, and (**d**) size of microplastics in pavement sediments in different functional zones of Beijing City.

The distribution of polymer types of microplastics in pavement sediments in different functional areas of Beijing is shown in Figure 5a, with PE, PP, PET, PA, and PS as the dominant polymers. In the commercial area, PP (26.3%) was the most abundant, followed by PE (25.1%), while PE occupied the highest percentage in each of the other four functional areas, with residential, transportation and scenic areas accounting for 35.0%, 30.1% and 33.8%, respectively. Previous studies have reported that, in 2017, expenditure on food delivery services in China reached RMB 200 billion, and the meal containers and plastic bags required by major online food-ordering platforms amounted to approximately 40 million items per day, or 14.6 billion items annually. Given that polypropylene (PP) and polyethylene (PE) are commonly used in food packaging materials, such large-scale consumption may be one possible reason for the relatively high proportions of PP- and PE-related microplastics observed in the present study [44,45]. PET is a prevalent material in the textile industry. Therefore, areas with high pedestrian traffic, such as transport zones and tourist spots, experience greater deposition of PET fibers from clothing abrasion, leading to its higher detected abundance in these locations.

#### 3.2.3. Color, Shape and Size of MPs

Trends in the color distribution of microplastics were evident in all five functional areas (Figure 5b). In residential areas, transparent microplastic (31.9%) was the most common color, followed by green (19.2%) and black (18.8%); transportation areas were dominated by green microplastic (28.4%), followed by black (25.1%), and blue (17.3%); scenic areas were dominated by green microplastic (22.9%), followed by black (22.1%), and transparent (17.1%); commercial areas were dominated by black microplastic (23.6%), followed by transparent (15.8%), green and red (both 13.8%). Transportation and scenic areas had a higher percentage of green microplastics, which may be related to some of the green plastic fibers used in construction. The higher proportion of black microplastics in transportation areas is likely linked to the predominance of a single pollution source: tire wear particles. While general anthropogenic litter is less common at these sites, the constant vehicle traffic generates substantial amounts of dark-colored tire debris [46]. In contrast, residential areas exhibited the highest percentage of transparent microplastics. This is likely attributable to the prevalence of clear plastics in common household goods and packaging, such as transparent bags and bottles; most of the colored microplastics in this study originated from the commercial area, possibly because bright colors contribute to the attractiveness of merchandise [47].

As can be seen in Figure 5c, Fiber microplastics dominate in residential, commercial, scenic and transportation areas, with 47.4%, 53.4%, 61.2% and 59.1%, respectively. The high percentage of fibrous microplastics in these areas may be related to the high level of human activity in these areas, resulting in a higher amount of leftover or discarded fibrous plastics, which are produced by shedding or breaking of fibrous garments, blankets, and other textiles [11,48], whereas in residential areas fibrous microplastics mainly originate from laundry wastewater [49]. This was followed by fragmentation, with 41.0%, 40.4%, 30.3% and 34.8% of microplastic fragments detected in residential, commercial, scenic and transportation areas, respectively. The percentage of film-like microplastics was the least in different functional zones, which is presumed to be mainly derived from the decomposition of packaging bags.

The size distribution of microplastics is shown in Figure 5d. Among all the size classifications, microplastics smaller than 500 µm accounted for the largest percentage (49.3%), while those with sizes in the range of 2–5 µm accounted for the smallest percentage (12.8%). Small-sized microplastics have also often dominated in previous studies [50], consistent with the findings of this study. Among the measured microplastics, the average sizes of microplastics in the five functional areas were 856.81 µm for residential areas, 931.72 µm for commercial areas, 1153.85 µm for scenic areas, and 1110.42 µm for transportation areas. The percentage of microplastics smaller than 500 µm was the highest in commercial and residential areas compared to the other types of functional areas, with 52.1% and 55.6%, respectively, due to intensive human activity in these areas, which generates plastic pollution that is broken down into smaller sizes in the environment and subsequently accumulates there [23].

### 3.3. Ecological Risk Assessment of MPs

Among the three ecological risk assessment methods applied, the pollution load index classified the degree of microplastic pollution in Beijing as Class I. Because the calculation results are greatly influenced by the background concentration value, and the *PLI* model only considers the quantitative risk of MPs, they cannot accurately reflect the differences in microplastic pollution in different loops and functional areas. The shape, color, and size of microplastics can alter the exposure pathways of microplastics to humans, thereby affecting their ecological risks [51]. Meanwhile, additives in plastic products also affect the toxicity of microplastics [52], and these factors should also be taken into account when assessing the potential ecological risks of microplastics [53]. In addition, the potential ecological risk of microplastics in sediments is increased by the fact that microplastics can enter organisms as carriers of other contaminants [52]. Hence, current assessment methodologies require further refinement to provide environmental managers with a more comprehensive understanding of the ecological risks posed by sediment-associated microplastics. Under the current data, the Polymer Risk Index (*PRI*) appeared to be more useful in identifying spatial and functional differences in potential ecological risk, as it incorporates both polymer hazard scores and their relative abundances, thereby more effectively reflecting spatial and functional differences in microplastic pollution. Nevertheless, the current *PRI*-based framework still relies on hazard scores covering only 55 polymer types [26], With the continuous emergence of new polymers, the scoring system must be expanded to ensure a more comprehensive evaluation of risks associated with diverse polymer types in sediments.

#### 3.3.1. Pollution Load Index

**Figure 6:**
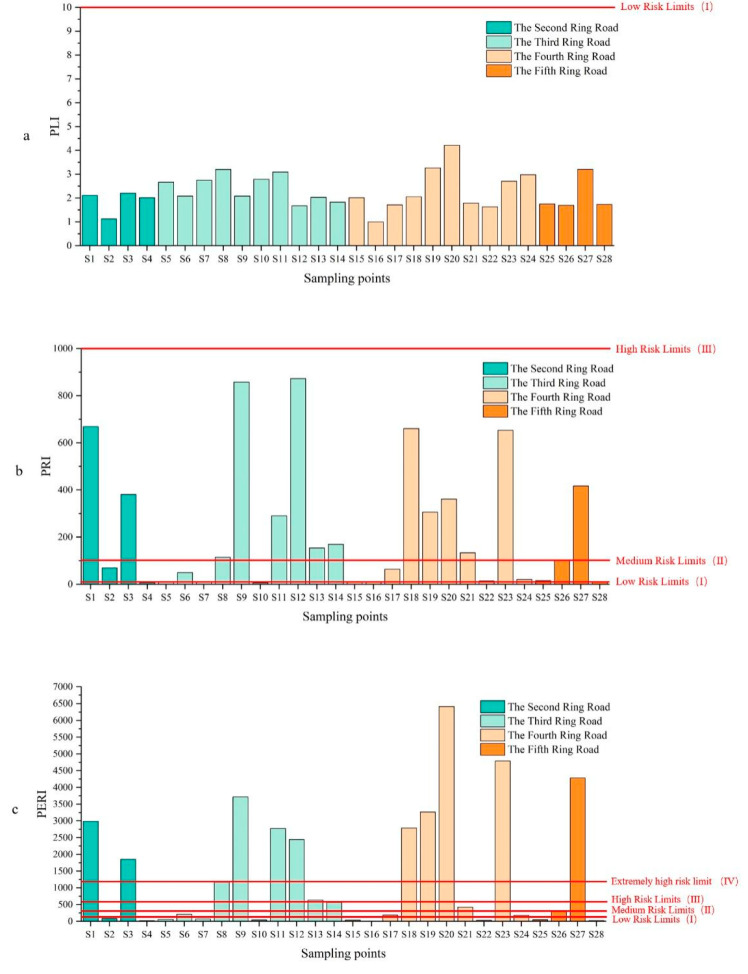
Microplastics in Beijing pavement sediments: (**a**) pollution load index, (**b**) polymer risk index, and (**c**) potential ecological risk index.

The risk of microplastic pollution in pavement sediments can be quantified by the pollution load index method [54]. In this study, the lowest value of microplastic abundance in pavement sediment (Wangheqiao bus stop, 170.55 items/kg) was selected as the background concentration (*C*_0_). According to the *PLI* assessment, all sampling sites were uniformly classified as Class I (Table A3 and Figure 6). Despite the uniform risk classification, the index values themselves revealed clear spatial discrepancies. Among the ring roads, the *PLI* decreased in the order of Chang’an Avenue (2.39) > Third Ring (2.36) > Fourth Ring (2.17) > Fifth Ring (2.01) > North–South Central Axis (1.91) > Second Ring (1.80). For functional zones, commercial areas exhibited the highest *PLI* (2.81), followed by residential (2.41), scenic (1.79), and transportation areas (1.78). It can be seen that the commercial area had the highest microplastic pollution load index, which is also consistent with the ranking of the commercial area in terms of abundance, among the different functional areas. However, it should be noted that the use of the minimum observed microplastic abundance as the background concentration (*C*_0_) may potentially underestimate the overall risk level. In addition, the *PLI* model considers only the quantitative risk of MPs and does not account for differences in ecological effects arising from polymer-specific toxicity [32]. Therefore, although the *PLI* remains limited in its ability to fully capture the overall ecological risk of microplastic pollution, it can still be used to provide a preliminary estimate of the pollution level in the study area.

#### 3.3.2. Polymer Risk Index

Assessment using the Polymer Risk Index (*PRI*) revealed a spectrum of contamination risks across Beijing’s pavement sediments. Overall, the majority of sites (15 of 28) were classified as Class III (high risk), alongside 8 Class II and 5 Class I sites (Table A4 and Figure 6). Spatially, this risk exhibited a clear gradient, decreasing from the city center outwards. The second ring road had the highest average *PRI*, while the fourth and fifth ring roads had lower average *PRI*s. The *PRI* of the Second Ring Road was 341.26, with one Class I, one Class II and two Class III risk locations; the *PRI* of the Third Ring Road was 287.38, with three Class I risk locations, one Class II risk location and six Class III risk locations; the *PRI* of the Fourth Ring Road was 181.33, with one Class I risk location, four Class II risk locations and five Class III risk locations; the *PRI* of the Fifth Ring Road was 181.79, with two Class II risk locations and two Class III risk locations.

The *PRI* values showed clear spatial heterogeneity across Beijing’s ring roads. The Second Ring Road had the highest *PRI* due to intensive commercial, residential, and traffic activities, with heavy use of single-use plastics and tire wear as major contributors. In contrast, the Third Ring Road displayed greater variability, with risk levels spanning Class I to Class III, consistent with its heterogeneous land-use functions that include transportation hubs, residential zones, and partial commercial areas, leading to uneven microplastic inputs [55], while the Fourth and Fifth Rings showed lower *PRI*s, mainly Class II–III, reflecting reduced commercial intensity, lower population density, and more frequent street cleaning that limited microplastic accumulation.

#### 3.3.3. Potential Ecological Risk Index

The Potential Ecological Risk Index (*PERI*) revealed a distinctly polarized risk landscape across Beijing, with classifications concentrated at the extremes: 10 sites fell into the lowest-risk category (Class I) and another 10 into the highest (Class V) (Table A5 and Figure 6). The analysis of *PLI*, *PRI*, and *PERI* identified commercial and high-traffic areas as ecological hotspots for microplastic pollution, in contrast to scenic areas, where the risk levels were relatively low. This difference likely due to more effective waste management practices, such findings are in line with previous studies, which emphasized the critical role of intensive anthropogenic activities in driving microplastic accumulation in urban commercial districts [56]. These results call for spatially targeted management strategies. High-risk commercial zones warrant prioritized interventions such as enhanced surface cleaning, stricter regulation of polymers like polyethylene (PE) and polypropylene (PP), and improved plastic waste management are essential. Therefore, integrating ecological risk assessments into urban governance frameworks is crucial for advancing non-point source pollution control and mitigating the long-term ecological threats of microplastic pollution.

#### 3.3.4. Correlation Analysis

**Figure A2:**
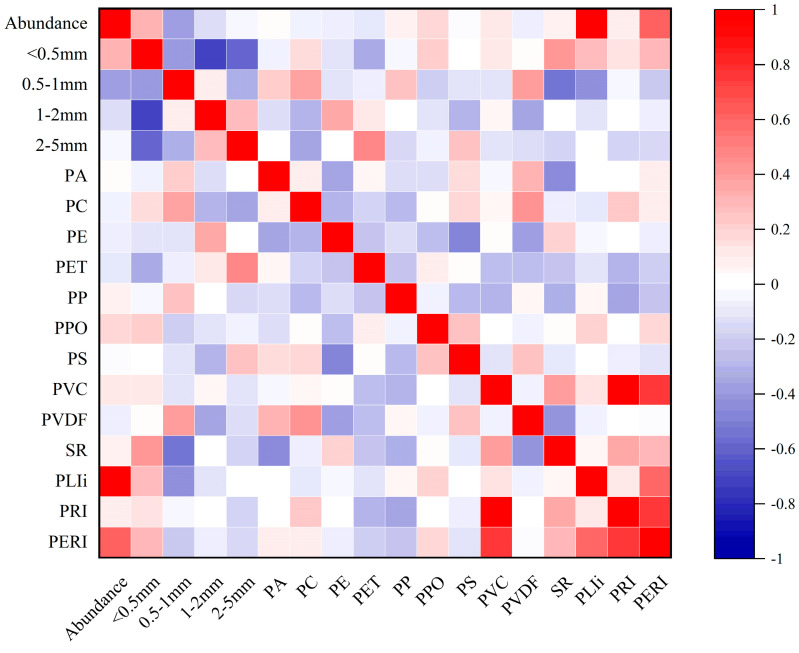
Correlation analysis of microplastic abundance, characteristics, and ecological risk indices.

Correlation analysis showed that microplastic abundance was positively correlated with all three risk indices (*PLI_i_*, *PRI*, and *PERI*), with particularly strong positive correlations with *PLI_i_* and *PERI* (Figure A2), indicating that increased microplastic accumulation generally corresponded to higher pollution load and potential ecological risk. Among the size fractions, particles smaller than 0.5 mm showed an overall positive correlation with the three risk indices, suggesting that small-sized microplastics may contribute more substantially to ecological risk. In terms of polymer composition, PVC exhibited positive correlations with all three risk indices, especially with *PRI* and *PERI*, indicating that high-risk polymers play an important role in driving ecological risk levels. In addition, SR was also positively correlated with *PRI* and *PERI*, suggesting that traffic-related particles may contribute to the ecological risk of microplastics in pavement sediments. Furthermore, the three risk indices were positively correlated with one another, with the strongest correlation observed between *PRI* and *PERI*, further highlighting the important role of polymer hazard characteristics in ecological risk assessment.

## 4. Conclusions

This study investigated the abundance and characteristics of microplastics in the pavement sediments of the north–south central axis, Chang’an Avenue, and its extension, and the major ring roads of Beijing. The average microplastic abundance was 960.9 items/kg, with the highest level observed in commercial areas (1505.7 items/kg). The main characteristics of microplastics were black (22.5%), fibrous (55.3%), small and medium-sized (10–500 µm, 47.0%), and made of PE (30.7%). This study found five Class I risk sites, eight Class II risk sites and 15 Class III risk sites, in its evaluation. This report clarifies the distribution of microplastic pollutants across different ring roads and functional areas in Beijing, with a view to enhance understanding of microplastic transport patterns in urban pavement sediments and providing a scientific basis for the management of runoff pollution. The development of sound management measures for microplastic pollution, based on the specific characteristics of microplastic pollution in a given region, is a priority for future research.

At present, ecological risk assessment methods for microplastics are still not sufficiently standardized or comprehensive, and further efforts are needed to establish a more scientific and unified risk assessment framework. Existing approaches, such as the Polymer Risk Index (*PRI*), remain constrained by limited polymer coverage and do not fully account for the complexity of environmental microplastics, including emerging polymer types, mixture effects, size-dependent risks, and vector effects. Therefore, future studies should focus on developing more robust and standardized assessment systems to improve the accuracy and comparability of microplastic risk evaluation across different environmental regions.

Another limitation of this study is that the sampling was conducted during a single campaign. Although the selected 28 sites provided broad spatial coverage across major ring roads and representative functional zones of Beijing, temporal variability was not addressed. Factors such as seasonal change, traffic intensity, precipitation, and street cleaning may influence the accumulation and distribution of microplastics in pavement sediments. Therefore, the findings of this study should be interpreted as representing the spatial characteristics of microplastics during the specific sampling period. Future studies with repeated or multi-seasonal sampling are needed to further strengthen the temporal representativeness and robustness of the conclusions.

## Data Availability

The original contributions presented in this study are included in the article. Further inquiries can be directed to the corresponding author.

## Appendix A

**Table A1:** Latitude and longitude of the road sediment sampling sites.

Location Number	Sampling Point Area	Affiliated Loop Area	Functional Area	Longitude (°)	Latitude (°)
S1	Gulou Street Subway Station, Xicheng District	2	Transportation area	116.40021	39.95460
S2	Fuxingmen Subway Station, Xicheng District	2	Transportation area	116.36376	39.91303
S3	Diplomatic Residence, Jianguomenwai, Dongcheng District	2	Residential area	116.44070	39.91536
S4	Yongdingmen Park, Dongcheng District	2	Scenic area	116.40550	39.88285
S5	Sidaokou North Street, Haidian District	3	Residential area	116.34850	39.97072
S6	Anhuaxili 1, Chaoyang District	3	Residential area	116.40060	39.97488
S7	Phoenix Gateway Shopping Center, Chaoyang District	3	Commercial area	116.46357	39.96688
S8	Haidian District Wanshousi Apartment	3	Residential area	116.31575	39.95506
S9	Gongzhufen Store, Town and Country Shopping Center, Haidian District	3	Commercial area	116.31675	39.91370
S10	China World Mall, Chaoyang District	3	Commercial area	116.46588	39.91567
S11	Longhu Beijing Lize Tianjie, Fengtai District	3	Commercial area	116.34105	39.87211
S12	Shilihe Bridge South Bus Station, Chaoyang District	3	Transportation area	116.46407	39.87105
S13	Hesheng Plaza, Fengtai District	3	Commercial area	116.40659	39.86343
S14	Zhenguosi North Street District, Fengtai District	3	Residential area	116.36182	39.85199
S15	National Olympic Sports Center, Chaoyang District	4	Scenic area	116.40019	39.99232
S16	Wangheqiao Bus Station, Chaoyang District	4	Transportation area	116.44414	39.99499
S17	Haidian Park, Haidian District	4	Scenic area	116.44414	39.99242
S18	Jiuxianqiao Eleventh Street District, Chaoyang District	4	Residential area	116.49788	39.96767
S19	Hua Xi LIVE Center, Haidian District	4	Commercial area	116.28925	39.91892
S20	Chaoyang District China Central Place Shopping Center	4	Commercial area	116.50199	39.91489
S21	Gongdaqiao Bus Station, Chaoyang District	4	Transportation area	116.49609	39.88264
S22	Nanyuan Forest Wetland Park, Fengtai District	4	Scenic area	116.40738	39.83856
S23	Xiaochun Subway Station, Chaoyang District	4	Transportation area	116.45502	39.83993
S24	Keyi Road Subway Station, Fengtai District	4	Transportation area	116.30381	39.83866
S25	Ecological Corridor Bridge in Chaoyang District, Olsen Park	5	Scenic area	116.39836	40.02912
S26	Beijing Shijingshan Laoshan City Recreation Park, Shijingshan District	5	Scenic area	116.21552	39.91781
S27	A1 Dingfuzhuang West Street, Chaoyang District	5	Residential area	116.56137	39.91520
S28	West Yushun Park, Daxing District	5	Scenic area	116.40822	39.77230

**Table A2:** Plastic Polymer Hazard Scale.

Polymer Name	Abridge	Risk Score	Risk Level
Polyethylene	PE	11	II
Polypropylene	PP	1	I
Polyethylene terephthalate	PET	4	II
Polystyrene	PS	30	II
Polyamides	PA	47	III
Synthetic rubber	SR	Nc	
Polyvinyl chloride	PVC	10,551	V
Polycarbonate	PC	1177	IV
Polyphenylene ether	PPO	400	III
Polyvinylidene fluoride	PVDF	Nc	

**Table A3:** Pollution loading coefficients, pollution loading indices, and risk levels of roadway sediments in Beijing, at each sampling point.

Sampling Point	Pollution Load Factor (*CF_i_*)	Pollution Load Index (*PLI_i_*)	Risk Level	Sampling Point	Pollution Load Factor (*CF_i_*)	Pollution Load Index (*PLI_i_*)	Risk Level
S1	4.47	2.11	I	S15	4.07	2.02	I
S2	1.27	1.13	I	S16	1.00	1.00	I
S3	4.87	2.21	I	S17	2.93	1.71	I
S4	4.07	2.02	I	S18	4.22	2.05	I
S5	7.11	2.67	I	S19	10.67	3.27	I
S6	4.33	2.08	I	S20	17.78	4.22	I
S7	7.56	2.75	I	S21	3.20	1.79	I
S8	10.22	3.20	I	S22	2.67	1.63	I
S9	4.33	2.08	I	S23	7.33	2.71	I
S10	7.78	2.79	I	S24	8.87	2.98	I
S11	9.56	3.09	I	S25	3.07	1.75	I
S12	2.80	1.67	I	S26	2.87	1.69	I
S13	4.13	2.03	I	S27	10.27	3.20	I
S14	3.33	1.83	I	S28	3.00	1.73	I

**Table A4:** Polymer Risk Index and risk levels of roadway sediments in Beijing, at each sampling point.

Sampling Point	*PRI*	Risk Level	Sampling Point	*PRI*	Risk Level
S1	669.26	III	S15	10.31	II
S2	69.53	II	S16	8.73	I
S3	381.44	III	S17	63.95	II
S4	6.79	I	S18	660.39	III
S5	8.56	I	S19	306.08	III
S6	49.60	II	S20	361.03	III
S7	9.06	I	S21	133.85	III
S8	114.48	III	S22	13.98	II
S9	858.05	III	S23	653.24	III
S10	5.63	I	S24	20.47	II
S11	290.60	III	S25	15.59	II
S12	872.95	III	S26	101.30	III
S13	154.02	III	S27	417.11	III
S14	169.67	III	S28	10.22	II

**Table A5:** Potential Ecological Risk Index and risk levels of roadway sediments in Beijing, at each sampling point.

Sampling Point	*PERI*	Risk Level	Sampling Point	*PERI*	Risk Level
S1	2989.37	V	S15	41.93	I
S2	88.07	I	S16	8.73	I
S3	1856.33	V	S17	187.60	II
S4	27.60	I	S18	2788.31	V
S5	60.89	I	S19	3264.80	V
S6	214.93	II	S20	6418.22	V
S7	68.44	I	S21	428.33	III
S8	1170.22	IV	S22	37.27	I
S9	3718.20	V	S23	4790.44	V
S10	43.78	I	S24	181.53	II
S11	2776.80	V	S25	47.80	I
S12	2444.27	V	S26	290.40	II
S13	636.60	IV	S27	4282.36	V
S14	565.56	III	S28	30.67	I

## References

[1] PlasticsEurope (2018). Plastics—The Facts 2017: An Analysis of European Plastics Production, Demand and Waste Data.

[2] Rochman C.M., Browne M.A., Halpern B.S., Hentschel B.T., Hoh E., Karapanagioti H.K., Rios-Mendoza L.M., Takada H., Teh S., Thompson R.C. (2013). Classify plastic waste as hazardous. Nature.

[3] Thompson R.C., Courtene-Jones W., Boucher J., Pahl S., Raubenheimer K., Koelmans A.A. (2024). Twenty years of microplastic pollution research—What have we learned?. Science.

[4] Rynek R., Tekman M.B., Veit-Köhler G., Wagner S., Reemtsma T., Jahnke A. (2025). Plastics from Surface to Seabed: Vertical Distribution of (Micro) plastic Particles in the North Pacific Ocean. Environ. Sci. Technol..

[5] Crawford C.B., Quinn B. (2017). 3-Plastic production, waste and legislation. Microplastic Pollutants.

[6] Carbery M., O’Connor W., Thavamani P. (2018). Trophic transfer of microplastics and mixed contaminants in the marine food web and implications for human health. Environ. Int..

[7] Soudavari A., Barari F., Ehsani E., Bonyadi Z., Davoudi M. (2025). Occurrence and health risk assessment of microplastics in beverages and ice packs. Sci. Rep..

[8] Patchaiyappan A., Dowarah K., Ahmed S.Z., Prabakaran M., Jayakumar S., Thirunavukkarasu C., Devipriya S.P. (2021). Prevalence and characteristics of microplastics present in the street dust collected from Chennai metropolitan city, India. Chemosphere.

[9] Kitahara K.-I., Nakata H. (2020). Plastic additives as tracers of microplastic sources in Japanese road dusts. Sci. Total Environ..

[10] Yuan D., Zhao L., Yan C., Zhou J., Cui Y., Wu R., Cui J., Wang J., Wang C., Kou Y. (2023). Distribution characteristics of microplastics in storm-drain inlet sediments affected by the types of urban functional areas, economic and demographic conditions in southern Beijing. Environ. Res..

[11] de Jesus Piñon-Colin T., Rodriguez-Jimenez R., Rogel-Hernandez E., Alvarez-Andrade A., Wakida F.T. (2020). Microplastics in stormwater runoff in a semiarid region, Tijuana, Mexico. Sci. Total Environ..

[12] Roychand R., Pramanik B.K. (2020). Identification of micro-plastics in Australian road dust. J. Environ. Chem. Eng..

[13] Dehghani S., Moore F., Akhbarizadeh R. (2017). Microplastic pollution in deposited urban dust, Tehran metropolis, Iran. Environ. Sci. Pollut. Res..

[14] Avio C.G., Gorbi S., Regoli F. (2017). Plastics and microplastics in the oceans: From emerging pollutants to emerged threat. Mar. Environ. Res..

[15] Ziajahromi S., Drapper D., Hornbuckle A., Rintoul L., Leusch F.D. (2020). Microplastic pollution in a stormwater floating treatment wetland: Detection of tyre particles in sediment. Sci. Total Environ..

[16] Xu P., Peng G., Su L., Gao Y., Gao L., Li D. (2018). Microplastic risk assessment in surface waters: A case study in the Changjiang Estuary, China. Mar. Pollut. Bull..

[17] Huang Z., Hu B., Wang H. (2023). Analytical methods for microplastics in the environment: A review. Environ. Chem. Lett..

[18] Tomlinson D.L., Wilson J.G., Harris C., Jeffrey D. (1980). Problems in the assessment of heavy-metal levels in estuaries and the formation of a pollution index. Helgol. Meeresunters..

[19] Zhou Y., Zhang Z., Bao F., Du Y., Dong H., Wan C., Huang Y., Zhang H. (2024). Considering microplastic characteristics in ecological risk assessment: A case study for China. J. Hazard. Mater..

[20] Bellasi A., Binda G., Pozzi A., Boldrocchi G., Bettinetti R. (2021). The extraction of microplastics from sediments: An overview of existing methods and the proposal of a new and green alternative. Chemosphere.

[21] Guo Z., Boeing W.J., Xu Y., Borgomeo E., Mason S.A., Zhu Y.-G. (2021). Global meta-analysis of microplastic contamination in reservoirs with a novel framework. Water Res..

[22] Li R., Yu L., Chai M., Wu H., Zhu X. (2020). The distribution, characteristics and ecological risks of microplastics in the mangroves of Southern China. Sci. Total Environ..

[23] Hoshyari E., Hassanzadeh N., Keshavarzi B., Jaafarzadeh N., Rezaei M. (2023). Characterization of microplastic, metals associated and ecological risk assessment in the topsoil of shiraz metropolis, south west of Iran. Chemosphere.

[24] Lithner D., Larsson Å., Dave G. (2011). Environmental and health hazard ranking and assessment of plastic polymers based on chemical composition. Sci. Total Environ..

[25] Huang Q., Liu M., Cao X., Liu Z. (2023). Occurrence of microplastics pollution in the Yangtze River: Distinct characteristics of spatial distribution and basin-wide ecological risk assessment. Water Res..

[26] Ranjani M., Veerasingam S., Venkatachalapathy R., Mugilarasan M., Bagaev A., Mukhanov V., Vethamony P. (2021). Assessment of potential ecological risk of microplastics in the coastal sediments of India: A meta-analysis. Mar. Pollut. Bull..

[27] Boni W., Arbuckle-Keil G., Fahrenfeld N. (2022). Inter-storm variation in microplastic concentration and polymer type at stormwater outfalls and a bioretention basin. Sci. Total Environ..

[28] Liu F., Vianello A., Vollertsen J. (2019). Retention of microplastics in sediments of urban and highway stormwater retention ponds. Environ. Pollut..

[29] Liu P., Shao L., Li Y., Jones T., Cao Y., Yang C.-X., Zhang M., Santosh M., Feng X., BéruBé K. (2022). Microplastic atmospheric dustfall pollution in urban environment: Evidence from the types, distribution, and probable sources in Beijing, China. Sci. Total Environ..

[30] Zhang M., Liu L., Xu D., Zhang B., Li J., Gao B. (2022). Small-sized microplastics (<500 μm) in roadside soils of Beijing, China: Accumulation, stability, and human exposure risk. Environ. Pollut..

[31] Li X., Liang R., Li Y., Zhang Y., Wang Y., Li K. (2021). Microplastics in inland freshwater environments with different regional functions: A case study on the Chengdu Plain. Sci. Total Environ..

[32] Du A., Li Y., Jian Q., Zhang K., Luo Y., Yan J., Du P., Power D.M., Li Y., Ma Y. (2025). Multidimensional characterization of microplastic pollution in subtropical urban soils: Combining geospatial analysis and polymer risk indexing. J. Hazard. Mater..

[33] Wang T., Niu S., Wu J., Yu J. (2022). Seasonal and daily occurrence of microplastic pollution in urban road dust. J. Clean. Prod..

[34] Polukarova M., Markiewicz A., Björklund K., Strömvall A.-M., Galfi H., Sköld Y.A., Gustafsson M., Järlskog I., Aronsson M. (2020). Organic pollutants, nano-and microparticles in street sweeping road dust and washwater. Environ. Int..

[35] Gu Y., Zhang X., Liu J., Deng J., Zhang Z., Tan C., Li H., Hu Y. (2025). Microplastics in road sediment of typical urban districts of Beijing: Characteristics and risk assessment. Process Saf. Environ. Prot..

[36] Phuong N.N., Poirier L., Lagarde F., Kamari A., Zalouk-Vergnoux A. (2018). Microplastic abundance and characteristics in French Atlantic coastal sediments using a new extraction method. Environ. Pollut..

[37] Zhao S., Zhu L., Wang T., Li D. (2014). Suspended microplastics in the surface water of the Yangtze Estuary System, China: First observations on occurrence, distribution. Mar. Pollut. Bull..

[38] Abbasi S., Keshavarzi B., Moore F., Turner A., Kelly F.J., Dominguez A.O., Jaafarzadeh N. (2019). Distribution and potential health impacts of microplastics and microrubbers in air and street dusts from Asaluyeh County, Iran. Environ. Pollut..

[39] Weber C.J., Opp C. (2020). Spatial patterns of mesoplastics and coarse microplastics in floodplain soils as resulting from land use and fluvial processes. Environ. Pollut..

[40] Fadare O.O., Okoffo E.D. (2020). COVID-19 face masks: A potential source of microplastic fibers in the environment. Sci. Total Environ..

[41] Jin X., Fu X., Lu W., Wang H. (2022). Fugitive release and influencing factors of microplastics in urbanized watersheds: A case study of the central area of Suzhou City. Sci. Total Environ..

[42] Fan Y., Zheng J., Xu W., Zhang Q., Chen N., Wang H., Qian X., Wang G. (2024). Spatiotemporal occurrence and characteristics of microplastics in the urban road dust in a megacity, eastern China. J. Hazard. Mater..

[43] Wang Q., Huang K., Li Y., Zhang Y., Yan L., Xu K., Huang S., Junaid M., Wang J. (2022). Microplastics abundance, distribution, and composition in freshwater and sediments from the largest Xijin Wetland Park, Nanning, South China. Gondwana Res..

[44] Yukioka S., Tanaka S., Nabetani Y., Suzuki Y., Ushijima T., Fujii S., Takada H., Van Tran Q., Singh S. (2020). Occurrence and characteristics of microplastics in surface road dust in Kusatsu (Japan), Da Nang (Vietnam), and Kathmandu (Nepal). Environ. Pollut..

[45] Deng Y., Lei K., An L., Liu R., Wang L., Zhang J. (2018). Countermeasurces on control of plastic litter and microplastic pollution. Bull. Chin. Acad. Sci..

[46] Bhavsar P.S., Chovatiya B.V., Kamble S.B., Gore A.H. (2024). Extraction and analysis of microplastics in the soil of Diamond City, Surat (Gujarat, India): Ecological risk, pollution indices, and greenness evaluation. ACS Agric. Sci. Technol..

[47] Belzagui F., Crespi M., Álvarez A., Gutiérrez-Bouzán C., Vilaseca M. (2019). Microplastics’ emissions: Microfibers’ detachment from textile garments. Environ. Pollut..

[48] Fu Z., Wang J. (2019). Current practices and future perspectives of microplastic pollution in freshwater ecosystems in China. Sci. Total Environ..

[49] Zhou G., Wang Q., Zhang J., Li Q., Wang Y., Wang M., Huang X. (2020). Distribution and characteristics of microplastics in urban waters of seven cities in the Tuojiang River basin, China. Environ. Res..

[50] Wang T., Zou X., Li B., Yao Y., Zang Z., Li Y., Yu W., Wang W. (2019). Preliminary study of the source apportionment and diversity of microplastics: Taking floating microplastics in the South China Sea as an example. Environ. Pollut..

[51] Wang Y., Zhou B., Chen H., Yuan R., Wang F. (2022). Distribution, biological effects and biofilms of microplastics in freshwater systems-a review. Chemosphere.

[52] Cao Y., Zhao M., Ma X., Song Y., Zuo S., Li H., Deng W. (2021). A critical review on the interactions of microplastics with heavy metals: Mechanism and their combined effect on organisms and humans. Sci. Total Environ..

[53] Feng Y., Chenglin L., Bowen W. (2019). Evaluation of heavy metal pollution in the sediment of Poyang Lake based on stochastic geo-accumulation model (SGM). Sci. Total Environ..

[54] Pan Z., Liu Q., Jiang R., Li W., Sun X., Lin H., Jiang S., Huang H. (2021). Microplastic pollution and ecological risk assessment in an estuarine environment: The Dongshan Bay of China. Chemosphere.

[55] Rakib M.R.J., Hossain M.B., Kumar R., Ullah M.A., Al Nahian S., Rima N.N., Choudhury T.R., Liba S.I., Yu J., Khandaker M.U. (2022). Spatial distribution and risk assessments due to the microplastics pollution in sediments of Karnaphuli River Estuary, Bangladesh. Sci. Rep..

[56] He B., Shi C., Chen B., Wu H., Goonetilleke A., Liu A. (2023). Occurrence and risk associated with urban road-deposited microplastics. J. Hazard. Mater..

